# Impact of Access Cavity Design on Fracture Resistance of Endodontically Treated Maxillary First Premolar: In Vitro

**DOI:** 10.1590/0103-6440202405676

**Published:** 2024-03-22

**Authors:** Anju Daniel, Abdul Rahman Saleh, Anas Al-Jadaa, Waad Kheder

**Affiliations:** 1 Department of Clinical Sciences, College of Dentistry, Ajman University, Ajman, United Arab Emirates; 2 Department of Clinical Sciences, College of Dentistry, Ajman University, Ajman, United Arab Emirates; Center of Medical and Bio-allied Health Sciences Research, Ajman University, Ajman, United Arab Emirates; 3 Department of Restorative Dentistry, College of Dental Medicine, University of Sharjah, Sharjah, United Arab Emirates.

**Keywords:** Ceramic, acid etching, silane, electrical current, adhesive bond

## Abstract

This study was designed to investigate the impact of access cavity designs on fracture resistance of endodontically treated maxillary first premolars. The study sample consisted of 72 intact maxillary first premolars, randomly divided into six groups (n = 12). A standardized proximal cavity preparation was prepared for all samples using standard bur. Groups I: control group with only standard proximal cavity and no endodontic access, group II: Truss access cavity, group III: Separated access to buccal and palatal canals without removal of dentine in between, group IV: Access to buccal and palatal canals with removal of dentine in between, group V: Traditional access cavity, group VI: Mesio-occlusal-distal cavity (MOD). For groups I and VI, only composite restoration was used to restore the proximal cavity, while for groups II- V, the access was prepared and endodontic treatment was performed on all teeth, then composite restoration was placed. The root canals were instrumented using nickel-titanium files, irrigated with sodium hypochlorite, and filled with AH plus sealer and gutta-percha using warm vertical condensation. All samples were then placed in an acrylic mold and underwent thermal aging for 10,000 cycles between 5 and 55°C. The samples were fixed in a universal testing machine with the long axis of the roots positioned at 20° to a load applied at a crosshead speed of 1 mm/min using a stainless steel semi-spherical indenter (Ø = 3 mm) until fracture occurred to determine the fracture resistance force in Newton. The normality test (Shapiro-Wilk) showed that data are normally distributed. Group II exhibited the highest mean fracture resistance, and group VI was the least likely to resist the fracture. No statistically significant differences between tested groups (p-value = 0.237). The MOD group showed a more unfavorable mode of fracture compared to other groups. No significant difference in fracture resistance between conservative and traditional access cavities. The missing marginal ridges, such as in MOD cavities played an important role in decreasing the fracture resistance of endodontically treated teeth.

## Resumo

O objetivo deste estudo foi investigar o impacto do design da cavidade de acesso na resistência à fratura de primeiros pré-molares superiores tratados endodonticamente. MÉTODOS: A amostra do estudo consistiu em 72 primeiros pré-molares superiores intactos, divididos aleatoriamente em seis grupos (n = 12). Um preparo padronizado da cavidade proximal foi realizado para todas as amostras usando broca padrão. Grupos I: grupo de controle com apenas cavidade proximal padrão e sem acesso endodôntico, grupo II: cavidade de acesso Truss, grupo III: acesso separado aos canais vestibular e palatino sem remoção de dentina entre eles, grupo IV: acesso aos canais vestibular e palatino com remoção de dentina entre eles, grupo V: cavidade de acesso tradicional, grupo VI: cavidade mesio-oclusal-distal (MOD). Para os grupos I e VI, apenas a restauração de compósito foi usada para restaurar a cavidade proximal. Já nos grupos II e V, o acesso foi preparado e o tratamento endodôntico foi realizado em todos os dentes e, em seguida, foi colocada a restauração de resina composta. Os canais radiculares foram instrumentados com limas de níquel-titânio, irrigados com hipoclorito de sódio e preenchidos com AH plus sealer e guta-percha usando condensação vertical quente. Todas as amostras foram então colocadas em molde de acrílico e submetidas a envelhecimento térmico por 10.000 ciclos entre 5 e 55°C. As amostras foram fixadas em uma máquina de teste universal com o eixo longo das raízes posicionado a 20° para uma carga aplicada a uma velocidade de cruzeta de 1 mm/min usando indentador semiesférico de aço inoxidável (Ø = 3 mm) até que ocorresse a fratura para determinar a força de resistência à fratura em Newton. RESULTADOS: O teste de normalidade (Shapiro-Wilk) mostrou que os dados são normalmente distribuídos. O grupo II apresentou a maior resistência média à fratura, e o grupo VI foi o menos propenso a resistir à fratura. Não houve diferenças estatisticamente significativas entre os grupos testados (p-valor = 0,237). O grupo MOD apresentou um modo de fratura mais desfavorável em comparação com os outros grupos. CONCLUSÕES: Não houve diferença significativa na resistência à fratura entre as cavidades de acesso conservador e tradicional. A ausência de cristas marginais, como nas cavidades MOD, desempenhou um papel importante na diminuição da resistência à fratura dos dentes tratados endodonticamente.

## Introduction

The success of root canal treatment is heavily reliant on seeing the canal orifices. Therefore, the primary function of access cavity preparation is to provide access for endodontic instruments to the root canal system. If adequate vision is not achieved, canals may be missed, increasing the likelihood of endodontic treatment failure ^1^. The significant loss of dental tissues due to irreversible caries and fractures has been recognized as a prevalent cause of root canal therapy failure ^2^. The purpose of access cavity design, aside from the carious affected area, is to preserve dentine by leaving a space between cavities so that the dentinal area provides the tooth with the needed strength ^3^. The amount of tissue removed, and the precise cavity layout created are thought to be strongly related to the fracture of these teeth ^4^. The more tissue eliminated, the weaker the remaining tooth structure becomes, as do tooth stiffness and fracture resistance. It is believed that trauma or fatigue failure from repeated stress overloading are the two main causes of root-filled teeth's susceptibility to fracture. Different types of restorations have been produced and advocated throughout the years for root-filled teeth ^5^. Making a restoration that could protect the remaining tooth structure from fracture under occlusal stress was always the clinical objective to make up for tissue loss. Therefore, there has been a significant shift in the medical field towards minimally invasive treatments. Minimal access preparation has been a popular subject in the endodontic field, and dentistry follows in its footsteps. The idea is to have an effective way to reduce the occurrence of post-treatment tooth fractures and support its goal of optimizing dentine tissue preservation ^6^.

The primary goal of a minimally invasive access cavity is to improve the mechanical stability and fracture resistance of the tooth, leading to long-term survival and function. The peri-cervical dentine and pulp chamber roof, which are located around 4 mm above and below the crestal bone, are believed to oversee transferring and balancing occlusal forces to the root (7, 8). As a result, researchers developed a novel cavity design to preserve as many of these features as possible. The pulp chamber ceiling being partially preserved, which would lessen the cusps' flexion, is the safest option to prevent destroying this structure ^7^. This is only possible with the help of modern technology. The foundation of the minimally invasive endodontic approach is conserving as much dentine as possible during access cavity preparation, maintaining optimal conditions for the longevity and function of endodontically treated teeth ^7^. Designs for minimally invasive access cavities carry greater risk in terms of how well the endodontic treatment works out. When the appropriate armamentarium is available, clinicians should carefully reconsider using minimally invasive access cavities in certain situations for routine endodontics ^9^. 

A traditional access cavity (TradAC) is defined as a cavity that aims to perform a complete unroofing of the pulp chamber, exposure of all pulp horns, and straight-line access to the root canals with coronally divergent walls and no undercuts to visualize the pulp chamber floor and all root canal orifices from the same visual angulation ^10^. The convenience form and extension for prevention of the access cavity are critical steps ^11^. However, due to enhanced illumination and magnification as well as the usage of narrow ultrasonic tips, either TradAC or conservative access cavity (ConsAC) no longer affects root canal detection ^12^. Only two studies have evaluated the impact of various access cavity designs on restoration methods, and they found that premolars with Ultra access cavity and premolars with TradAC had fewer voids in the composite restoration than those prepared with ConsAC ^13^ ^,^ ^14^. However, the best restorative material is natural tooth substance, and smaller-volume cavities are easier for both the operator and the patient to treat ^15^.

Silva and co-workers concluded that there is no balance between clinical and experimental outcomes. Since samples were not adequately standardized and processed, no model has been verified to truly rank the materials and processes ^6^. The knowledge of different access cavity designs is of the utmost importance, as the access cavity constitutes an integral and crucial part of endodontic treatment ^16^. Considering these contradictory findings and methodological problems, better-controlled and well-designed ex vivo investigations employing novel methodological techniques are necessary to elucidate the effect of access cavity preparation on the fracture resistance of teeth. The main objective of this study was to investigate the impact of access cavity designs on fracture resistance of endodontically treated maxillary first premolars. 

## Materials and methods

### Study design

The local research ethics committee at Ajman University approved the study. A total of 72 recently extracted, intact maxillary first premolars were collected from patients between 15 and 25 years of age, who were undergoing orthodontic treatment. The teeth were examined using a dental operating microscope (DOM) (CJ-Optik, Flexion, Synka, Germany) to ensure they were free of caries, cracks, restorations, or any other defects. The calculus and soft tissue were removed from the selected teeth by using an ultrasonic scaler (Suprasson® P5 Newtron SATELEC; ACTEON, Merignac, France). Periapical radiographs were taken to ensure the presence of two roots, and completely formed apices, and to rule out the presence of previous endodontic treatment or calcifications. Throughout the various phases of the research, extracted teeth were maintained in a 0.9% saline solution to prevent dehydration ^17^. To limit the effect of shape and size variances on the outcomes, homogenous groups were formed based on the averages of tooth dimensions ^11^. A caliper (Calipretto CR, Renfert, Hilzingen, Germany) was used to measure the mesiodistal and buccolingual dimensions of each tooth at the level of the tooth's cervical third ^18^ and tabulated in an Excel sheet (Microsoft Excel, Microsoft Corporation, 2022). The average buccolingual (BL) dimension of the crown was measured as 8.1 ± 0.2 mm and the mesiodistal (MD) dimension as 4.8 ± 0.1 mm ^19^. 

### Sample size calculation

The sample size was calculated using the statistical power analysis software G*Power (3.1.9.3 for Macintosh; Heinrich Heine, Universität Dusseldorf, Dusseldorf, Germany) ^20^. The ANOVA: Fixed effects, omnibus, one-way test was selected from the F test. Accordingly, for the analysis with α=0.05 and 90% testing power and considering an effect size = 0.5, the total sample size was 72 (12 samples per group (n=12).

### Samples preparation

**Figure 1:**
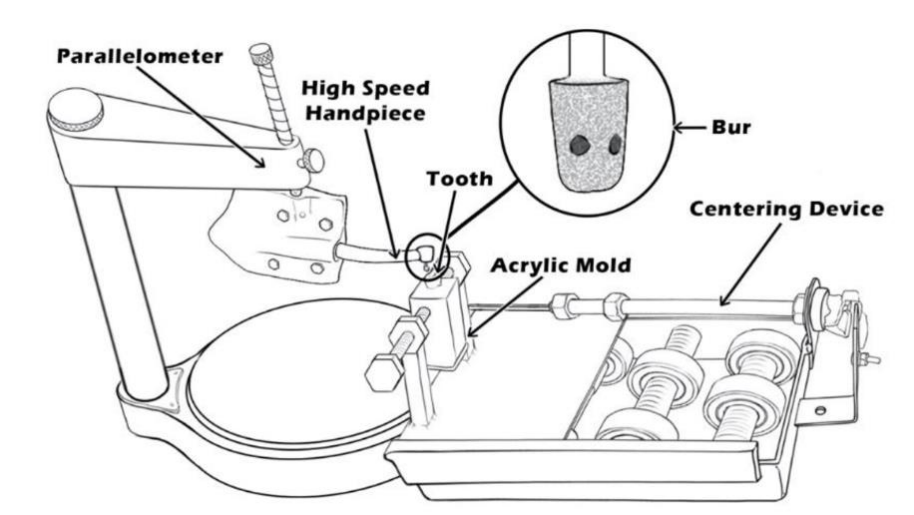
The schematic drawing represents the centering device parallelometer.

A standardized distal proximal cavity preparation was initially made with a high-speed handpiece and a diamond bur - 845KRD.314.025 (Komet, Germany), for all the teeth to simulate the caries removal procedure. To make a consistent proximal cavity with the same dimensions (2.5mm wide and 4mm deep) for all samples, the tooth was held with fast-setting bite registration impression material (Zhermack hydrorise monophase, Germany) in a rectangular acrylic mold and fastened in a custom-made centering device that moves slowly forward when it is switched on (can be interchanged clockwise or anticlockwise) towards the handpiece. The handpiece with the bur was held with the help of the VG 1N Degussa Parallelometer (Degussa, Frankfurt, Germany) during the preparation of the standardized cavity (Figure 1). All endodontic access cavities were accomplished using a high-speed round bur - 801XL-316-012 (GZ instrument, Germany); then the root canal procedure and final composite restoration were done.

### Samples grouping and access cavity design

**Figure 2:**
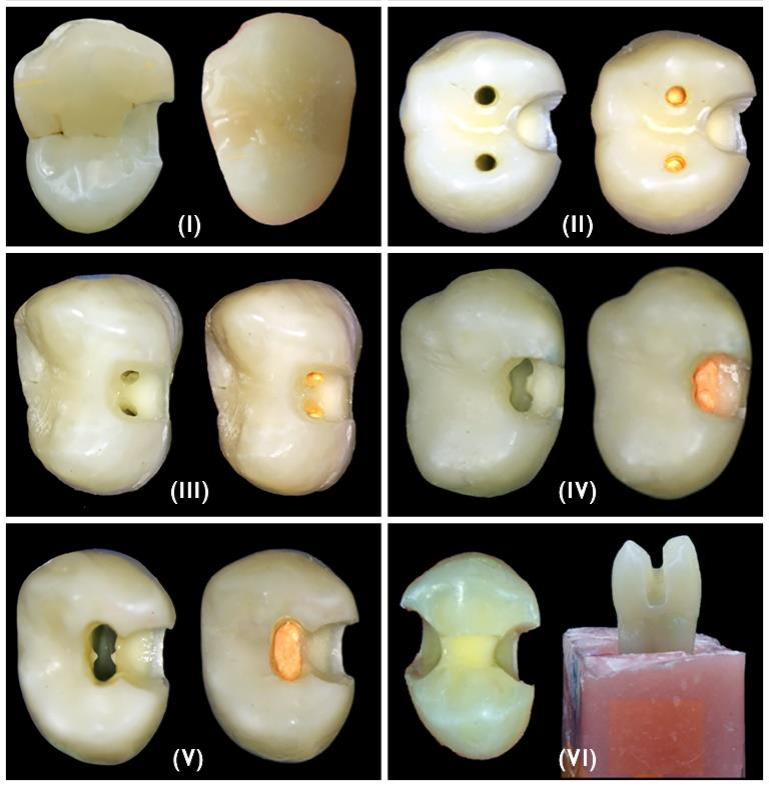
Types of endodontic access cavity design: Group I: Proximal cavity; Group II: Truss access cavity; Group III: A standard proximal cavity with separated buccal and palatal access through the proximal cavity without removal of dentine in between the canals; Group IV: A standard proximal cavity with both buccal and palatal access without the presence of dentin in between them; Group V: A standard proximal cavity with TradAC; Group VI: Mesio-occlusal-distal cavity.

To simulate the clinical conditions, each tooth was positioned, before the endodontic treatment, on a phantom head - Frasaco maxillary jaw (Frasaco Franz Sachs, Tettnang, Germany). One operator prepares the root canals under magnification using a CJ-Optik operating microscope (Flexion Advanced; CJ-Optik, Germany). The samples were assigned to 6 groups (n = 12) based on the type of endodontic access cavity design (Figure 2).

Group I: Control group, only proximal cavity using the standard bur, without endodontic access cavity, then the cavity restored with composite resin. 

Group II: Truss access cavity, a standard proximal cavity with direct, two-separated, straight access to the buccal and palatal root canals. 

Group III: A standard proximal cavity with separated buccal and palatal access through the proximal cavity without removal of dentine in between the canals. 

Group IV: A standard proximal cavity with both buccal and palatal access without the presence of dentin in between them. The dentin in between the canals was removed with an Endo Z bur (Dentsply Maillefer, Ballaigues, Switzerland). 

Group V: A standard proximal cavity with TradAC. 

Group VI: Mesio-occlusal-distal cavity (MOD) without endodontic access, then the cavities restored with composite resin. 

### Endodontic treatment

The root canals were negotiated with size 8 or 10 K files (Dentsply Maillefer, Ballaigues, Switzerland) to the required working length. A size 15-K file was used to establish the glide path. Then, the root canals were instrumented using the WaveOne reciprocating system (Dentsply Sirona, Ballaigues, Switzerland) up to primary for both buccal and palatal root canals ^11^. The root canals were intermittently irrigated throughout instrumentation with 1.3% sodium hypochlorite (NaOCl) in between each file. WaveOne Gold absorbent paper points were used to dry the root canals. Root canals were filled by the warm vertical condensation technique. The final restoration was done using composite resin.

### Simulation of periodontal ligament and thermocycling

Before the fracture resistance test, the periodontal ligament and alveolar bone were simulated with a thin layer of wax and self-curing acrylic resin in a custom-made metal holder. Every sample was immersed in the wax for the prescribed amount of time and temperature. The difference (0.2 to 0.3 mm) in the diameter of the root before and after dipping in the wax was then verified by measuring the thickness of the root using a digital caliper. Nonetheless, a wax carver was used to remove extra wax. As soon as the acrylic began to polymerize, the root was removed from the mold to ensure that the wax was not affected by the polymerization of acrylic ^21^. All specimens were kept in a 0.9% saline solution in a humid incubator at 36°C in between all the procedures and before thermocycling (Incubator I, Memmert, Schwabach, Germany). Then, in a computerized thermocycling unit (SD Mechatronik GmbH, Feldkirchen-Westerham, Germany), the specimens were thermocycled simultaneously for 10,000 cycles, which was equivalent to one year of clinical service between two temperature extremes of 5 and 55° C (dwell time: 30 seconds, pause time: 13 seconds).

### Fracture resistance testing

**Figure 3:**
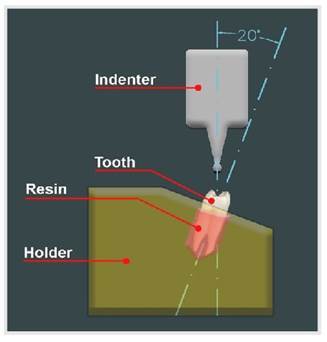
The schematic drawing represents the universal testing machine with the indenter positioned against the lingual slope of the buccal cusp.

All samples were inspected after the thermocycling, and none of the samples showed visible deformation. The fracture resistance of each sample was then measured using a universal testing machine (universal testing machine, M350-5CT, Testometric, Rochdale, UK) and evaluated a lingual slope of the buccal cusp (Figure 3). A compressive force of 1 mm/min was applied vertically until the final fracture occurred. The fracture load was measured in Newton. Fracture modes were classified as follows: Favorable: when the failures were above the acrylic resin level, indicating that the fracture site was above the bone level and was restorable. Unfavorable: where failures extended beyond the level of the acrylic resin, indicating that the fracture site was below the bone level and was difficult to restore or completely non-restorable. 

### Statistical analysis

The data was tabulated in Microsoft Excel, Microsoft Corporation, 2022, and the statistical analysis was performed with SPSS Software (IBM SPSS Statistics for Windows, Version 27.0. Armonk, NY: IBM Corp. Released 2020). A normality test (Shapiro-Wilk) indicates that the data on fracture resistance is normally distributed. A one-way analysis of variance (ANOVA) was used to examine the difference in fracture resistance between the groups. Statistically significant if the p-value is less than 0.05. To determine failure modes and the number of samples with favorable or unfavorable fractures, the Chi-square test was performed.

## Results

**Figure 4:**
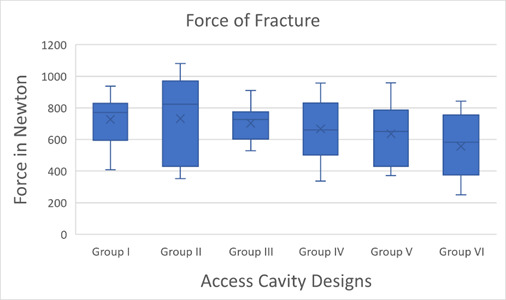
Box plot showing the mean, interquartile range, and force of fracture in Newtons for the study groups with different access cavity designs.

**Figure 5:**
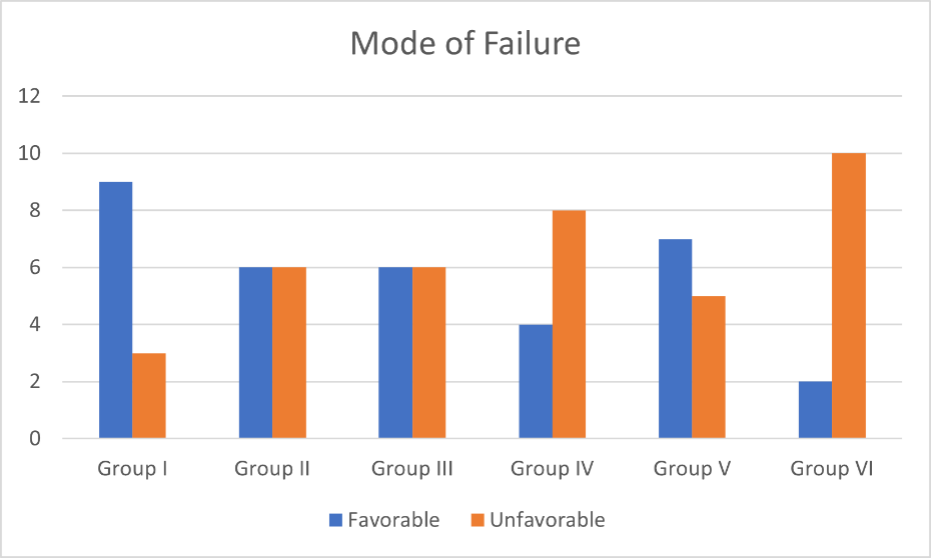
Failure modes of study groups characterized as favorable and unfavorable tooth fracture.

The finding showed that the fracture resistance force in group II (Truss access cavity) was the highest among all groups, with the mean force reaching up to 732.1 N and a standard deviation of ±265.3. On the other hand, the lowest fracture resistance was in group VI (MOD), with a mean force of 556.5 N and a standard deviation of ±200.8. The fracture resistance forces for groups I, III, IV, and V were ranked as follows from highest to lowest: group I (standard proximal cavity), group III (access from proximal cavity for buccal and palatal canal (separated), group IV (access from proximal cavity for buccal and palatal canal (joined), and group V (proximal cavity along with traditional endodontic access cavity), which showed a mean of 727.4 N, 702.7 N, 666.7 N, and 636.2 N and a standard deviation of ±179, ±107.7, ±186, and ±200.9 respectively. Besides, the results of ANOVA showed that there were no statistically significant differences between the tested experimental groups p-value = 0.237. (Figure 4). The results showed that MOD groups had significantly more unfavorable modes of failure compared to the other groups (p-value = 0.031). There was no significant difference in the mode of failure between the different designs of access cavity (p-value = 0.66), however, the control group with only the proximal cavity had a favorable mode of failure (Figure 5).

## Discussion

This study aimed to investigate the impact of access cavity designs on fracture resistance of root canal-treated teeth. The findings showed that there were no significant differences in fracture resistance between the study groups (P > 0.05); and this finding was in line with more than one publication in the same field ^22^ ^,^ ^23^ ^,^ ^24^ ^,^ ^25^ ^,^ ^26^. The influence of marginal ridge absence in the fracture resistance of the root canal-treated teeth was also included in the present study. Interestingly, the results showed that a study group with the MOD cavity preparation had the lowest mean fracture resistance and more unfavorable fracture compared to other study groups. This supports previous studies ^27^ ^,^ ^28^ which showed that the maximum thickness of axial tooth structure at the crown margins is required to resist fracture and the proximal ridges serve as supporting components of the posterior teeth's dental structure. However, some studies stated that marginal ridge does not decrease the fracture strength of root-filled teeth ^29^ ^,^ ^30^.

The preservation of sound tooth structure is crucial for the survival of teeth that have undergone root canal treatment ^18^. This was reflected in our project's findings, which confirmed that fracture resistance was correlated to both, access cavity design and the amount of reduced tooth structure. However, the access cavity itself is not the reason for the decrease in tooth fracture resistance, but the increase in the amount of tooth structure reduction during access cavity preparation may reduce the tooth fracture resistance. This was in accordance with findings by Reeh and co-workers, who showed that access cavity and root canal treatment appear to have only a minor effect on the tooth, with a 5% reduction in relative stiffness, compared with a 20% reduction in tooth stiffness due to restorative procedures for an occlusal restoration and a 63% reduction if both marginal ridges were lost in a MOD preparation ^31^. To minimize the mechanical failure of endodontically treated teeth, proximal caries-driven access that eliminates the occlusion contact areas from the tooth/restoration interface is technically achievable ^32^. 

The selection of the maxillary premolar in this study was related to the aesthetic concerns and lateral occlusal forces that this tooth may be subjected to during functional and parafunctional habits. The cusp fracture is reported to be more concentrated in root-filled maxillary premolars ^33^ ^,^ ^34^. The normal biting force is 222-445 N (average 322.5 N), while during clenching, the occlusal force can reach 520-800 N (average 660 N) ^35^. In addition, maxillary premolars with thin roots in the mesiodistal dimension are more likely to experience longitudinal root fractures ^36^. Furthermore, according to Schwartz and Robbins, premolars are subjected to lateral forces of a more detrimental nature than molars ^37^.

A study by Shahrbaf and co-workers found that endodontically treated maxillary premolars with MOD cavities had the lowest fracture resistance values (489.66 ±149.45 N) ^38^. This was in accordance with our study findings, as the fracture resistance was 556.58 ±200.80 N for the group of samples with MOD cavities, and without endodontic access cavities. The mean fracture resistance value for a study group with a MOD cavity was considerably lower than that of the control group with a DO cavity. Linn and Messer found that root canal-treated teeth with MOD cavities were substantially damaged due to the loss of supporting elements such as the pulp chamber roof and marginal ridges. Regardless of the kind of endodontic access cavity, the loss of both the mesial and distal ridges showed severely reduced tooth strength ^39^.

The correlation between the residual coronal dentine structure and fracture resistance of root canal-treated teeth was also reflected in a study by Ibrahim et al., (2016), who showed that tooth wall loss, particularly that of the mesial and distal marginal ridges, significantly reduces tooth fracture strength compared to the influence of access cavity design ^40^. These findings are consistent with a study by Sorrentino et al. (2007) ^41^. This indicates that the strategic placement of the remaining tooth structure may be more important than its actual volume. Corsentino and co-workers found that the fracture resistance of intact teeth is decreased by the endodontic access cavity design and the loss of one or two walls. The fracture strength of treated teeth, which was weakened by the removal of two marginal walls, was unaffected by using the access cavity designs (ConsAC or TradAC) ^24^.

In conclusion, all modifications in the design of the access cavity used in this study did not decrease the fracture resistance compared to TradAC and were not statistically different. When compared to its conventional counterpart, the conservative endodontic access cavity may increase the tooth's resistance to fracture, but not to a statistically significant level ^42^. Besides, there is no significant difference in fracture resistance between samples with various designs of access cavities. The presence of a marginal ridge is important to increase fracture resistance, so a maximum effort should be made to preserve marginal ridge structure since it significantly increases the fracture resistance of endodontically treated teeth. 

Clinically, it is essential to minimize the removal of healthy tooth structure while providing adequate access for effective cleaning, shaping, and obturation of the root canals. However, it is crucial to strike a balance between adequate access and preserving tooth strength to prevent post-treatment fractures. Our study showed very important clinical relevance by investigating the impact of access cavity design on the fracture resistance of the root canal-treated tooth.

Currently, it is advised to avoid a conservative approach and instead advocate for standard access cavity procedures. Magnification is ideal for conservative access cavities, but not all doctors have access to it. The process of locating the canal orifices, delivering irrigation efficiently, preventing iatrogenic harm by biomechanical preparation, and achieving superior obturation is faster and more predictable with a conventional access cavity ^43^.

Furthermore, the study had limitations as it did not use Micro-CT or CBCT, which is important to decrease anatomical bias in sample selection and distribution by matching samples based on root canal volume, surface area, configuration, pulp chamber, and dental hard tissues. Besides, the study did not use the chewing simulator to study the effect of simultaneous thermal and mechanical aging, which may add more clinical value to the findings.
